# Case Report: Dual-lumen microcatheter-facilitated wiring technique to correctly access a protruded aorto-ostial stent: a case series

**DOI:** 10.3389/fcvm.2025.1467926

**Published:** 2025-03-05

**Authors:** Seok Hyun Kim, Kook Jin Chun

**Affiliations:** Division of Cardiology, Department of Internal Medicine and Research Institute for Convergence of Biomedical Science and Technology, Pusan National University Yangsan Hospital, Pusan National University School of Medicine, Yangsan, Republic of Korea

**Keywords:** coronary artery stent, in-stent restenosis, percutaneous coronary intervention, myocardial infarction, acute coronary syndrome

## Abstract

**Background:**

Percutaneous coronary intervention (PCI) through the aorto-ostial coronary stent that is protruding into the aorta remains a technical challenge because of the poor coaxial alignment of the guiding catheter and the inability to advance the guidewire into the distal vessel through the stent's central lumen. In this article, we introduce a dual-lumen microcatheter–facilitated wiring technique performed on two patients to overcome this difficulty.

**Case summary:**

The first case was a 75-year-old man who presented with chest pain. He was diagnosed with an unstable angina, and coronary angiography showed near-total in-stent occlusion of the previously placed stent protruding into the aorta. Despite several attempts, the guidewire passed through the side strut of the stent instead of the central stent lumen. Thus, we placed the tip of the microcatheter proximally to the side strut, outside the stent. Then, a second wire was passed through the central lumen successfully. After confirming the wire's position via intravascular ultrasound, we inflated a drug-eluting balloon, subsequently obtaining a successful angiographic result. The second case was a 78-year-old woman diagnosed with non-ST segment elevation myocardial infarction. Coronary angiography revealed tight stenosis at the ostial left anterior descending artery with a previous stent deployed from the left main to the circumflex artery. Owing to the excessive overhanging stent into the aorta, the wire could not be advanced into the stent's central lumen. However, with the facilitation of a dual-lumen microcatheter, a second wire successfully passed through the stent's central lumen. Finally, the patient received a successful PCI with a stent.

**Conclusion:**

A dual-lumen microcatheter–facilitated wiring technique may be useful in overcoming wiring difficulty caused by the excessive protrusion of an aorto-ostial stent into the aorta.

## Introduction

Performing percutaneous coronary intervention (PCI) in patients with protruding aorto-ostial stent may be technically challenging because of a poor coaxial alignment of the guiding catheter and the inability to insert a wire through the central stent lumen (1). Several techniques, including a double-wire technique, balloon-assisted technique, snare technique, side-strut sequential ballooning technique, and guide extension catheter-facilitated side-strut stenting, have been proposed to treat this challenge. However, these methods have some limitations, such as frequent failure and procedure-related complications (2). Herein, we describe two cases of chest pain that necessitated technically challenging PCI for complex lesions with excessive stent protrusion into the aorta. The two cases were intervened by a novel dual-lumen microcatheter (DLC)-facilitated wiring technique with a successful angiographic result.

## Case presentation

### Case report 1: the right coronary artery

**Figure 1:**
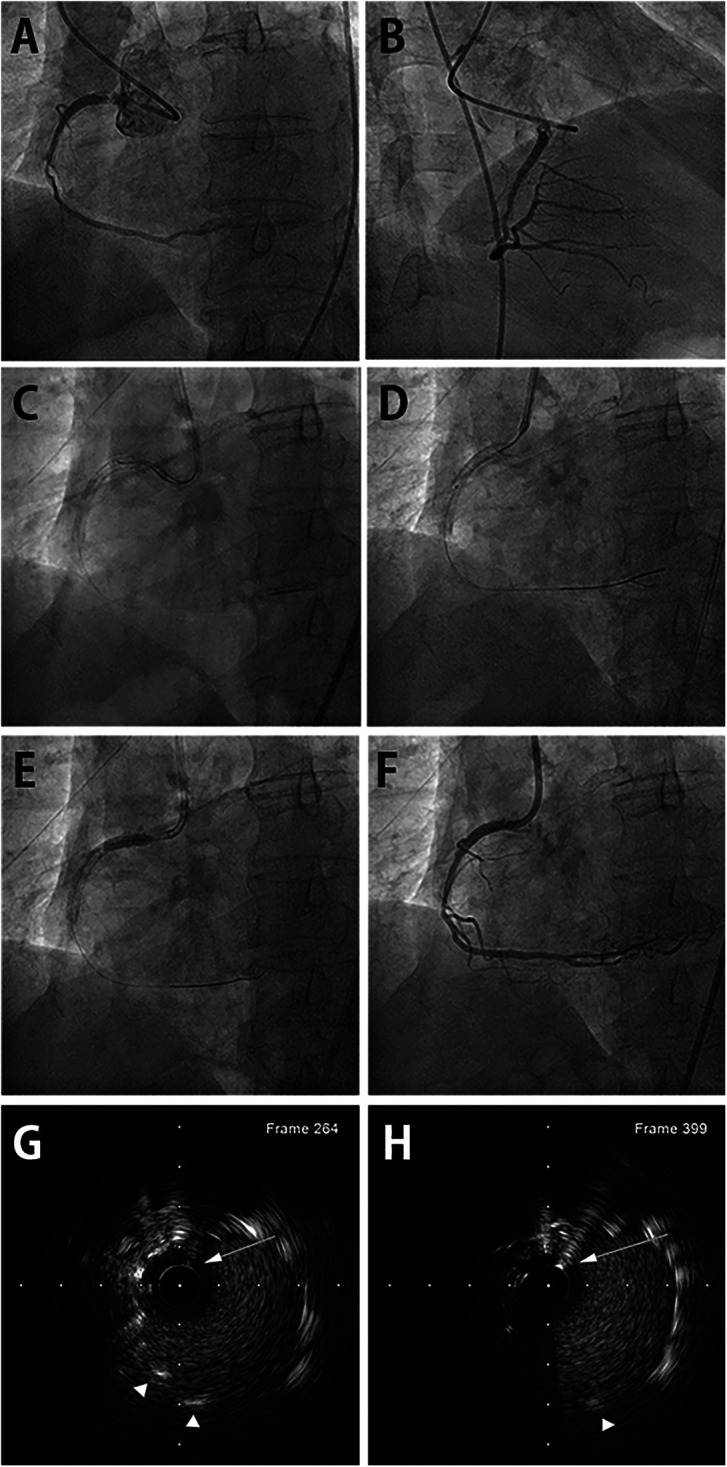
DLC-facilitated aorto-ostial wiring technique in the RCA. **(A)** Coronary angiography from the left anterior oblique view shows in-stent restenosis of the previously placed aorto-ostial RCA stent. **(B)** Non-selective angiography from the right anterior oblique view also reveals the in-stent restenosis, alluding challenging cannulation. **(C)** The first wire is inserted in the RCA through the stent-side strut. The second wire successfully enters the central stent lumen facilitated by DLC. **(D)** The wire's central position is confirmed using an intravascular ultrasound catheter. **(E)** The drug-eluting balloon is inflated after proper lesion preparation. **(F)** Final coronary angiography shows a good angiographic result with coaxially engaged guiding catheter. **(G)** IVUS image revealing luminal position of the second wire in the mid portion of the protruding stent outside the coronary artery. First wire was retracted before IVUS imaging. **(H)** IVUS image showing luminal position at the very proximal end of the protruding stent. DLC, dual-lumen microcatheter; RCA, right coronary artery; IVUS, intravascular ultrasound. (Arrows indicate second wire. Arrowheads indicate stent strut.)

A 75-year-old man presented with chest pain diagnosed as unstable angina and previously underwent multiple PCIs with stents. Coronary angiography revealed tight in-stent restenosis of the ostial right coronary artery (RCA). Fluoroscopy revealed that the previous ostial RCA stent was excessively protruding into the aorta. The RCA was engaged using a Judkins Right 3.5 guiding catheter. Passing the wire through the central column was extremely difficult because the guiding catheter was unstable. After several attempts, the SION blue (Asahi Intecc, Aichi, Japan) wire could only cross through a side strut of the previous stent and advance deeply into the distal RCA. Furthermore, the double-wire technique using a second guidewire failed to pass through the stent's central lumen. After we loaded Sasuke DLC (Asahi Intecc, Aichi, Japan) onto the wire and placed the DLC tip in the proximity of the side strut, the guiding catheter was slightly withdrawn away from the aortic wall. Ultimately, we achieved stable coaxial alignment of the guiding catheter a few millimeters away from the previous stent. A second SION (Asahi Intecc, Aichi, Japan) wire successfully accessed the central stent lumen through the over-the-wire port of the DLC, as confirmed by intravascular ultrasound (IVUS). It took 22 min from the introduction of DLC to the confirmation of the proper position of the second wire by IVUS. A 3.5 × 15 mm drug-eluting balloon was inflated at high pressure after proper balloon angioplasty preparation. The final angiography revealed excellent results without procedure-related complications (Figure 1).

### Case report 2: the left main coronary artery

**Figure 2:**
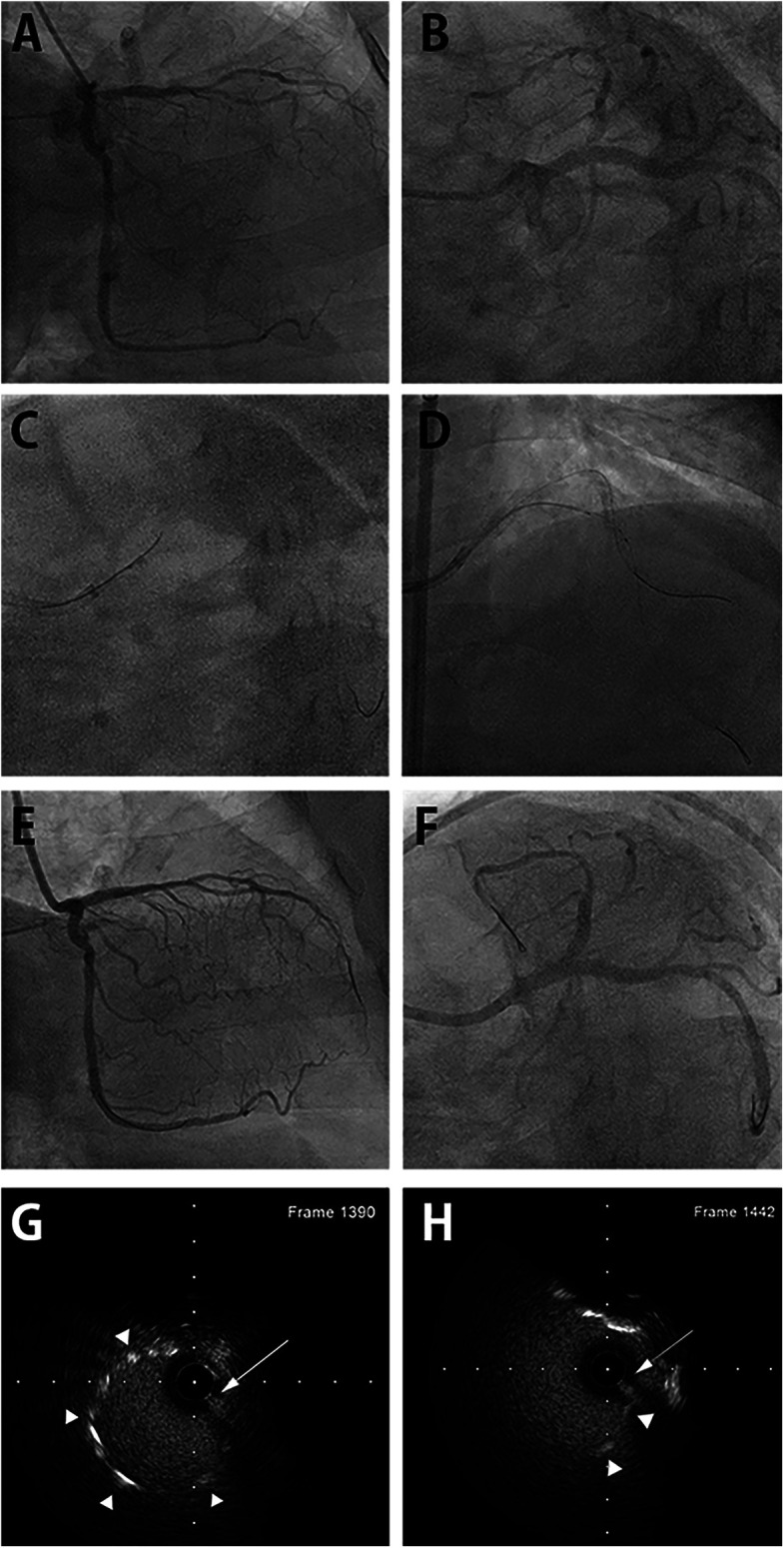
DLC-facilitated aorto-ostial wiring technique in the left main coronary artery. **(A)** Coronary angiography with a misaligned catheter from the right anterior oblique caudal view demonstrates a tight ostial stent in the left anterior descending artery and a patent stent spanning from the left main to the left circumflex artery. **(B)** The left anterior oblique caudal view exhibits the same finding, with the catheter tip placed next to the protruding aorto-ostial stent. **(C)** DLC is loaded onto the first wire and anchored at the protruding stent strut. The second wire enters the central stent lumen. **(D)** The wire's central position is confirmed by IVUS. **(E)** The final angiography from the right anterior oblique caudal view after stenting shows acceptable angiographic results with proper coaxial engagement of the guiding catheter. **(F)** Angiography from the left anterior oblique caudal view demonstrates a well-expanded stent with the coaxial position of the guiding catheter. **(G)** IVUS image revealing luminal position of second wire in the mid portion of the protruding stent outside the coronary artery. First wire was retracted before IVUS imaging. **(H)** IVUS image showing luminal position at the very proximal end of protruding stent. DLC, dual-lumen microcatheter; IVUS, intravascular ultrasound. (Arrows indicate second wire. Arrowheads indicate stent strut.)

A 78-year-old woman presented with chest pain and elevated cardiac biomarkers. She experienced myocardial infarction with PCI from the left main to the circumflex coronary artery and RCA at another hospital five years ago. Coronary angiography demonstrated severe stenosis of the ostial left anterior descending artery crossed by the previous stent. The left main portion of the stent was protruding deeply into the aorta. We then selected a Judkins Left 3.5 guiding catheter. However, the initial SION blue wire failed to pass through the stent's central lumen but entered the left circumflex artery through the side strut of the stent. Using a Sasuke DLC loaded onto the SION blue wire, we successfully advanced a second SION wire into the stent's central lumen through the DLC's over-the-wire port, and its position was confirmed by IVUS. It took 6 min from employing the DLC to the confirmation of central stent lumen position of the wire by IVUS. Subsequently, PCI with stent was performed successfully (Figure 2).

## Discussion

**Figure 3:**
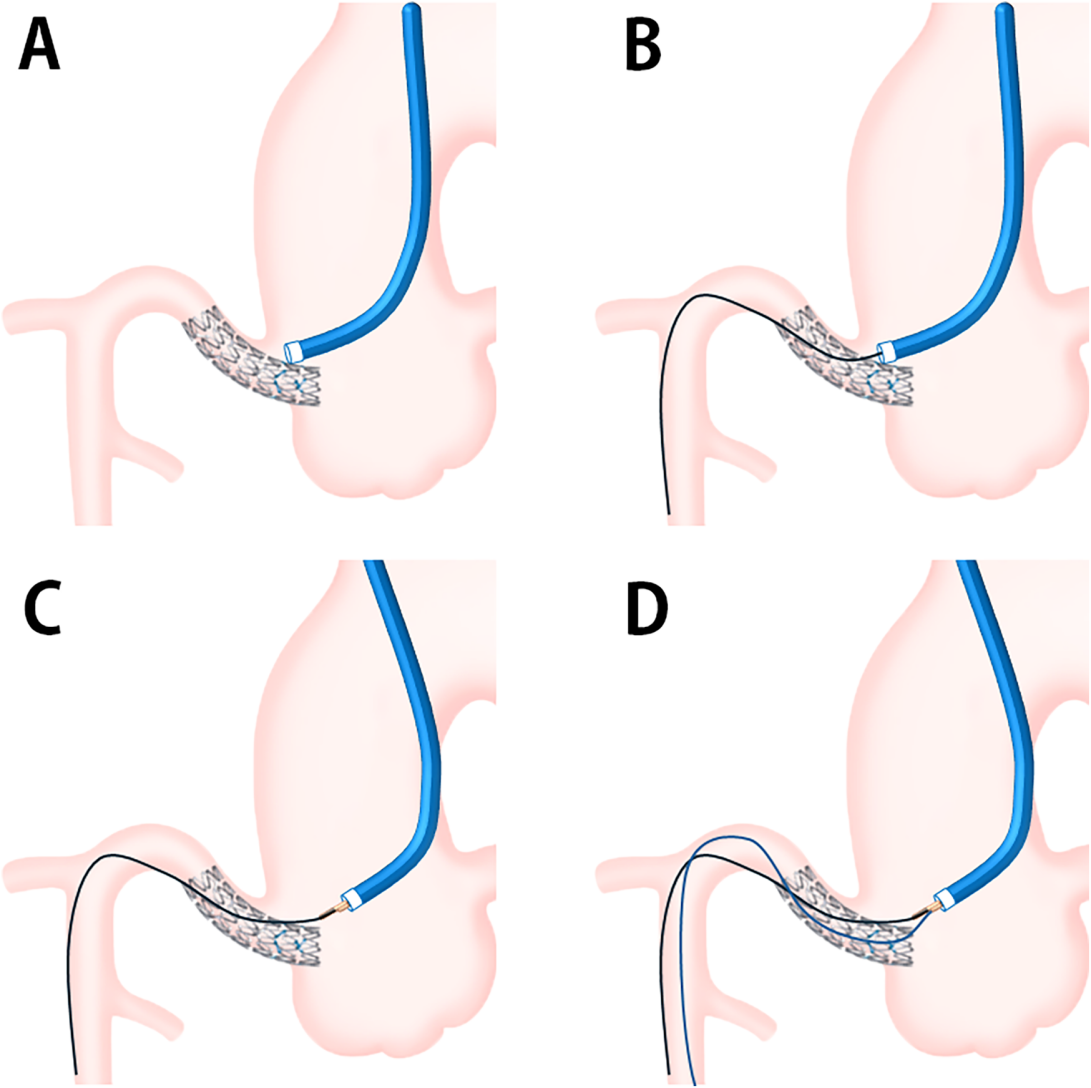
Schematic presentation of DLC-facilitated wiring technique. **(A)** Coaxial cannulation to the protruding aorto-ostial stent segment is challenging. **(B)** The first wire is advanced across the stent strut and deeply into the coronary artery. **(C)** DLC is loaded onto the first guidewire and placed on or near the protruding stent segment, providing sufficient support for guiding catheter manipulation to achieve coaxial access. **(D)** The second guidewire enters the central stent lumen through the over-the-wire port. DLC, dual-lumen microcatheter.

The aorto-ostial stent often protrudes into the aorta and more so after proximal optimization or aortic flaring, which is shown to elongate the stents (3). Repeat PCI on previously stented aorto-ostial lesions is extremely challenging and is associated with a higher risk for procedure-related complications. The DLC-facilitated wiring technique is a new approach to successfully pass through the stent's central lumen in patients with a protruding prior stent in the aorto-ostial location. DLC has been used to facilitate PCI for bifurcation lesion and chronic total occlusion (CTO). In bifurcation lesion, DLC is helpful for side-branch wiring because of the angulated side branches when conventional wiring has failed. DLC may also be useful for patients requiring a parallel wire technique during antegrade CTO PCI. The main concept of DLC is that the first wire acts as a guide through the rapid-exchange port and supports the second wire through the over-the-wire port that can be manipulated to find the target lumen (4). In DLC-facilitated wiring technique, we insert the first wire to the cell formed by the stent's strut and advance it deeply into the distal vessel; meanwhile, the DLC is positioned close to the stent, providing ample support. Subsequently, we manipulate the guiding catheter to create a slight distance from the stent ostium and align it coaxially. The DLC tip need not be anchored directly to the side strut; its proximity to the stent is usually sufficient. Then, we pass a second wire into the stent's central lumen through the over-the-wire port of the DLC. Once the second wire successfully traverses the central stent lumen, we remove the DLC and the first wire. Confirming the position of the second wire using intracoronary imaging is crucial, and if unsuccessful, we repeat the process (Figure 3).

Several aorto-ostial wiring techniques, including a double-wire technique (1), balloon-assisted access technique (2), double-guide snare technique (5), side-strut sequential ballooning technique (6), and guide-extension–facilitated side-strut stenting (7), have been proposed to address this challenge. However, we need to be aware of the caveats of each method. In the double-wire technique (1), a single wire must provide all the support for the catheter while being manipulated for alignment; consequently, system instability and prolonged PCI time often occur. The balloon-assisted access technique (2) offers enhanced support with minimal risk of stent deformation, avulsion, or extraction. However, continual torquing of the wire for central lumen wiring may cause entanglement and intertwining with the first wire and balloon catheter, thereby compromising the procedure. Moreover, the double-guide snare technique (5) requires double arterial access. Given that the side-strut sequential ballooning technique (6) and guide-extension–facilitated side-strut stenting technique (7) are performed through the side strut, the risk of stent deformation increases; hence, they are used only for extremely protruding stents as the last resort. Other potential complications, such as wiring failure, stent avulsion, deformation, and extraction, should also be considered (7, 8). The DLC-facilitated aorto-ostial wiring technique provides sufficient support for creating a gap between the catheter and the stent while avoiding device intertwining in the shaft of the guiding catheter as well as stent deformation and avulsion, even during prolonged procedures. It applies to both aorto-ostial RCA and left main coronary artery with protruding stents, as shown in this report. Furthermore, our experience demonstrates that this technique substantially reduces the time required to wire the stent's central lumen. After passing the second wire, intracoronary imaging confirmation of its proper location is mandatory. While we utilized the Sasuke DLC, other DLCs designed for the same purpose from different companies may also offer similar performance benefits. The difference between the recently reported DLC and the floating-wire technique by Wong et al. (9) is that DLC is anchored at or near the protruding stent segment, providing augmented support and coaxiality at the same time until central lumen wiring is achieved; therefore, it does not serve solely as a tool to fine-tune the coaxiality over the dummy wire.

**Figure 4:**
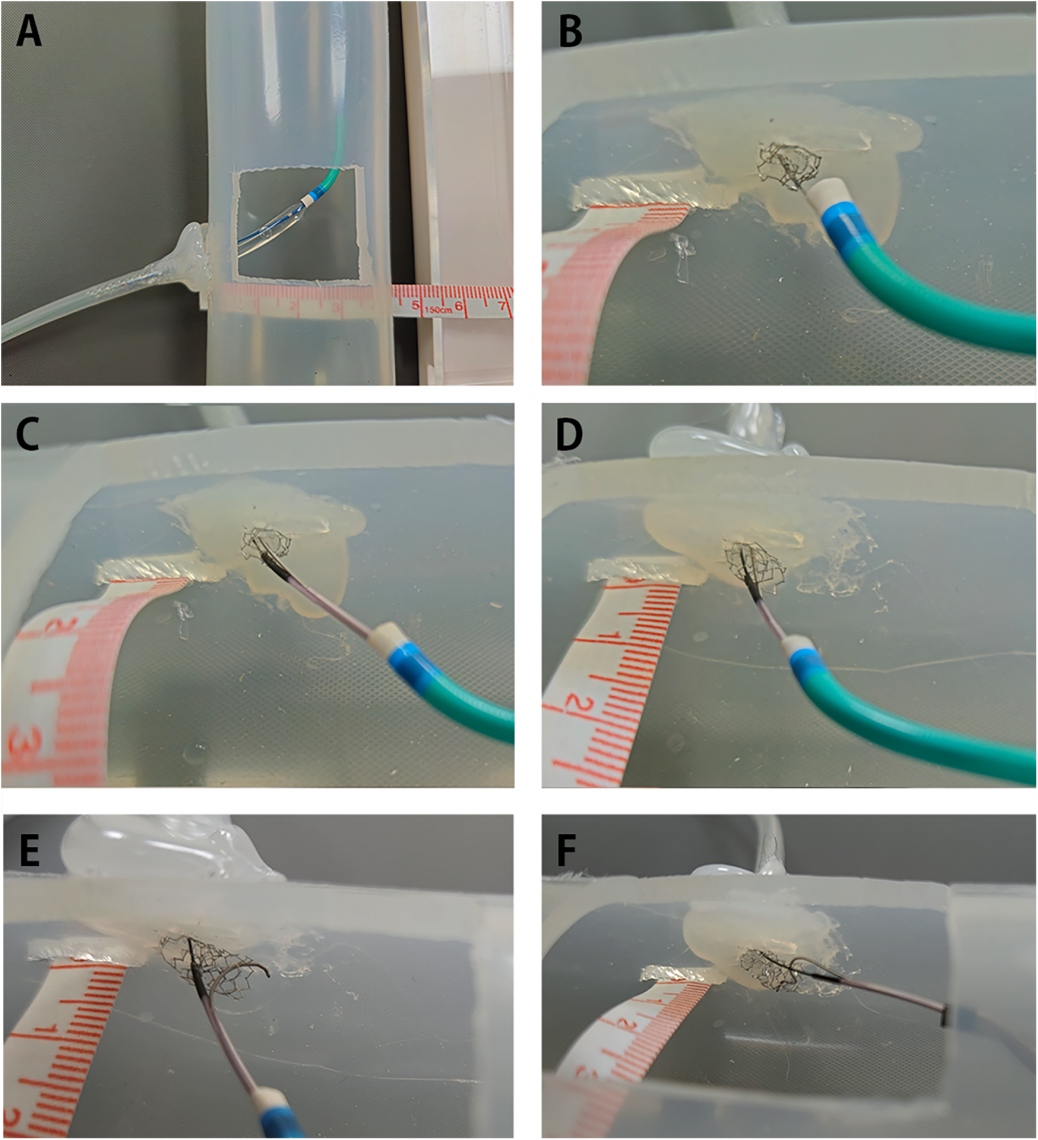
Bench test of wiring a protruding aorto-ostial stent. Bench test showing DLC-facilitated wiring for 2, 4, and 6 mm stent protrusion. Note that the first wire in each test crossed the stent cell from below. The procedural times in this bench test do not include the time spent on location confirmation using intracoronary imaging. The guide catheter used in **(A–E)** was a 6 Fr Judkins Right. Video files for **(C–F)** are available. **(A)** A silicone tube with a diameter of 4.0 mm, representing the right coronary artery, was attached to a silicone tube with a diameter of 40 mm, simulating the aorta. A rectangular window was cut to allow clear visualization of the wiring process. A 4-mm Synergy (Boston Scientific, Natick, MA, USA) or Orsiro (Biotronik AG, Bülach, Switzerland) drug-eluting stent was deployed to achieve the intended length of protrusion with sequential flaring in each test. **(B)** Aggressive pushing of the guide catheter against the stent easily crushed the stent, complicating subsequent procedures. **(C)** Successful wiring into the central stent lumen when the stent protruded 2 mm into the aorta. It took approximately 3 min to correctly direct the wire, guided by visual observation. **(D)** Successful wiring into the central stent lumen when the stent protruded 4 mm into the aorta. It took approximately 10 min to correctly direct the wire, guided by visual observation. **(E)** Even with meticulous manipulation guided by visual observation, the stent column tended to tilt upward, losing coaxial alignment with the second wire when the stent protruded 6 mm into the aorta. Repeated attempts failed to pass the wire correctly after 30 min. **(F)** Exchanging the guide catheter for a 6 Fr Amplatz Left improved alignment but also failed to pass the second wire when the stent protruded 6 mm into the aorta. Although the second wire successfully entered the central lumen a few times, it frequently lost its correct position, escaping through the side cell. Attempts to use stiff or polymer-coated soft wires also failed. DLC, dual-lumen microcatheter.

There are several limitations to our technique. First, aggressively pushing the system, such as the DLC and guide catheter, may crush the stent and should be avoided. Second, although we successfully managed to place the wire properly in the two cases mentioned, it may not always be possible to pass the wire into the central stent lumen within a limited time frame. Running intracoronary imaging with each attempt and restarting the technique if the second wire crosses the side cell will prolong the procedure and may be inappropriate in certain clinical situations. Third, exaggerated stent protrusion (more than a few millimeters) may hinder the DLC from keeping the guide catheter aligned with the axial stent opening. We performed a bench test to explore this issue (Figure 4; Supplementary Video S1). Initially, we hypothesized that utilizing the Sasuke DLC would enable us to correctly wire the central lumen up to 6-mm protrusion, as the distance between the rapid exchange port and the over-the-wire port is 6.5 mm, which should direct the second wire from the proximal stent lumen. We were able to successfully position the wire for up to 4 mm of protrusion within an acceptable time frame. However, when the protrusion exceeded 4 mm, the stent lumen lost coaxial alignment, and it was no longer possible to wire through it from the proximal beginning. Therefore, the cut-off length for successfully wiring from the proximal stent lumen appears to depend on the protrusion length itself rather than the properties of the DLC. For excessive protrusions greater than 4 mm, creating a new central lumen through the side cell may be the only practical option. Since it is difficult to accurately recognize the protruding stent length without prior PCI records or information, determining when to stop attempting to wire the central lumen can help reduce unnecessary fluoroscopy exposure and procedure time—an issue that our study could not address. Finally, our report is based on only two cases, and comparisons regarding whether DLC-facilitated wiring is superior to other techniques in terms of safety and procedural time cannot be made. Further studies with a larger sample size are needed to explore the appropriate strategy for specific scenarios. However, the purpose of our study is to report the utility of easily applying familiar instruments to challenging PCI situations.

## Conclusions

In conclusion, the DLC-facilitated wiring technique is useful in overcoming the difficulty of wiring the aorto-ostial stent that protrudes excessively into the aorta while minimizing the risk of wire intertwining, stent strut deformation, and procedural failure.

## Data Availability

The original contributions presented in the study are included in the article/Supplementary Material; further inquiries can be directed to the corresponding author.

## Supplementary material

Supplementary Video S1 Bench test of wiring a protruding aorto-ostial stent. Clip 1 2-mm protrusion. Clip 2 4-mm protrusion. Clip 3 6-mm protrusion (JR, failed). Clip 4 6-mm protrusion (AL, failed).
